# Treatments for sleep disturbances in individuals with acquired brain injury: A systematic review

**DOI:** 10.1177/02692155211014827

**Published:** 2021-05-20

**Authors:** Louise Pilon, Nikita Frankenmolen, Dirk Bertens

**Affiliations:** 1 Rehabilitation Centre Klimmendaal, Arnhem, The Netherlands; 2 Donders Institute for Brain, Cognition and Behaviour, Radboud University, Nijmegen, The Netherlands

**Keywords:** Sleep disturbances, acquired brain injury, interventions

## Abstract

**Objective:**

To systematically review the evidence on the treatments of sleep disturbances in individuals with acquired brain injury.

**Data sources:**

PubMed, Embase, Web of Science, and PsycINFO were searched from inception to January 2021.

**Review method:**

Eligibility criteria were (1) participants with mild to severe acquired brain injury from traumatic brain injury and stroke (⩾three months post-injury), (2) individuals aged 16 years and older, (3) participants with self-reported sleep disturbances, (4) controlled group studies and single case (experimental) studies, and (5) interventions aimed at treatment of sleep disturbances. Two researchers independently identified relevant studies and assessed their study quality using the revised Cochrane assessment of bias tool (RoB 2.0) and the risk-of-bias in N-of-1 trials (RoBiNT) scale.

**Results:**

The search yielded 655 records; 11 studies met the inclusion criteria and were included, with a total of 227 participants (207 individuals with traumatic brain injury, 20 stroke patients). Two studies included pharmacological therapy, six studies examined the effects of cognitive behavioral therapy and three studies investigated alternative interventions such as acupuncture.

**Conclusion:**

Although there was heterogeneity in the study quality of the included studies, their outcomes suggest that cognitive behavioral therapy is recommended as treatment of choice for improving sleep in individuals with acquired brain injury, especially for patients with mild to severe traumatic brain injury. Future research should examine the effects of cognitive behavioral therapy in more high-quality randomized controlled designs.

## Introduction

Sleep disturbances are commonly reported following acquired brain injury and have a negative impact on functioning.^1^ In the context of this review, acquired brain injury includes traumatic brain injury and stroke. It is estimated that fifty to seventy percent of individuals with traumatic brain injury (50%) or stroke (67%) in the chronic phase of recovery suffer from sleep disturbances, which is much higher than the incidence in the general population.^2–4^ Up to a third of the persons with acquired brain injury experience insomnia, which is defined as an inability to sleep, particularly associated with problems of falling asleep and maintaining sleep.^5–7^ Since many studies did not diagnose sleep complaints as sleep disorders, this review focuses on sleep disturbances refering to sleep problems that occur at night and are characterized by the inability to initiate and maintain sleep. Several studies have reported a poorer sleep quality and a reduced sleep efficiency (ratio of time spent asleep compared with time spent in bed) due to more frequent awakenings at night.^8–10^ Following acquired brain injury, sleep disturbances can exacerbate other injury-related symptoms (e.g. cognitive functioning and fatigue) and have a negative impact on recovery.^11,12^ The high prevalence of sleep disturbances, its negative outcomes and also the persisting nature of sleep disturbances stress the need for effective sleep interventions for individuals with acquired brain injury.

Available treatment options for sleep disturbances in the general population could be divided into pharmacological treatment, cognitive behavioral therapy (CBT) or other, alternative interventions (e.g. acupuncture).^13^ Although clear guidelines exist for treatment of sleep disturbances, little research has been done on the effects of these treatments in people with acquired brain injury. As a result, sleep disturbances are often not part of standard care within the longer-term outpatient rehabilitation of patients with acquired brain injury.^3,14^

Recently, a systematic review on non-pharmacological treatment for insomnia showed beneficial effects of cognitive behavioral therapy in patients with acquired brain injury.^15^ However, a general overview including both pharmacological and non-pharmacological treatments for sleep disturbances following acquired brain injury is lacking. Our review aims to systematically review the evidence on the treatments of sleep disturbances in individuals with acquired brain injury. Specific aims are to evaluate the effectiveness of pharmacological treatment, cognitive behavioral therapy and other interventions in sleep disturbances and to discuss the implications of the findings for clinical practice, based on the study quality of the included studies.

## Methods

A systematic review was performed to evaluate interventions for sleep disturbances in individuals with acquired brain injury. The present systematic review followed the Preferred Reporting Items for Systematic Reviews and Meta-Analyses guidelines (PRISMA)^16^ to search, extract and evaluate the interventions. An electronic search was conducted in the following international databases: Web of Science, PubMed, Embase, and PsycINFO with the latest search performed on January 11, 2021. The search was not limited by any restrictions regarding publication date or language. The search strategy was developed in collaboration with a clinical librarian and was constructed using the following themes: sleep disturbance, acquired brain injury and intervention. Identification of key search terms was followed, using synonyms of the search terms. Key search terms were combined using the Boolean “OR”; themes were combined using the Boolean “AND”. The search strategy was adjusted accordingly to optimize the specificity of the search. The search strategy of each database is included in Supplemental Appendix 1.

Studies were included based on the following criteria: (1) participants with mild to severe acquired brain injury following a traumatic brain injury or a stroke (⩾three months post-injury); (2) participants aged 16 years and older; (3) participants with self-reported sleep disturbances; (4) (randomized) controlled group studies and single case experimental studies; and (5) interventions aimed at treatment of sleep disturbances. Self-reported sleep disturbances encompass difficulties in initiating and maintaining sleep. Single case studies are defined as experimental, controlled interventions studied in a single case. Articles were excluded if they (1) were review articles or meta-analyses and (2) gray literature.

Two independent reviewers (LP, NF) determined eligibility criteria and systematically screened the records by titles and abstracts with respect to inclusion and exclusion criteria. Of the selected articles, full-texts were reviewed to verify that they met the criteria. Lack of agreement between the reviewers on this was settled through discussion.

Data extraction was performed by two independent researchers (LP, DB). A data extraction table was created, summarizing the most relevant data. The following information was extracted for each intervention study: authors, year of publication, study type, study design, method, sample (number of participants, type of acquired brain injury), intervention, primary outcome measure, findings, maintenance of effects, and study quality. A third researcher was consulted when consensus was not reached.

Methodological quality of the included studies, including risk of bias, was assessed by two independent reviewers (LP, NF), using guidelines specified for each research design. Randomized group studies were evaluated using the revised Cochrane assessment of bias tool (ROB 2.0 tool). The risk-of-bias in N-of-1 trials (RoBiNT) scale was used for the assessment of single case studies. The percentage of agreement between reviewers was 87% in terms of rating the methodological quality. Consensus with scoring was reached through discussion. Any lack of consensus was discussed with a third researcher.

## Results

**Figure 1.**
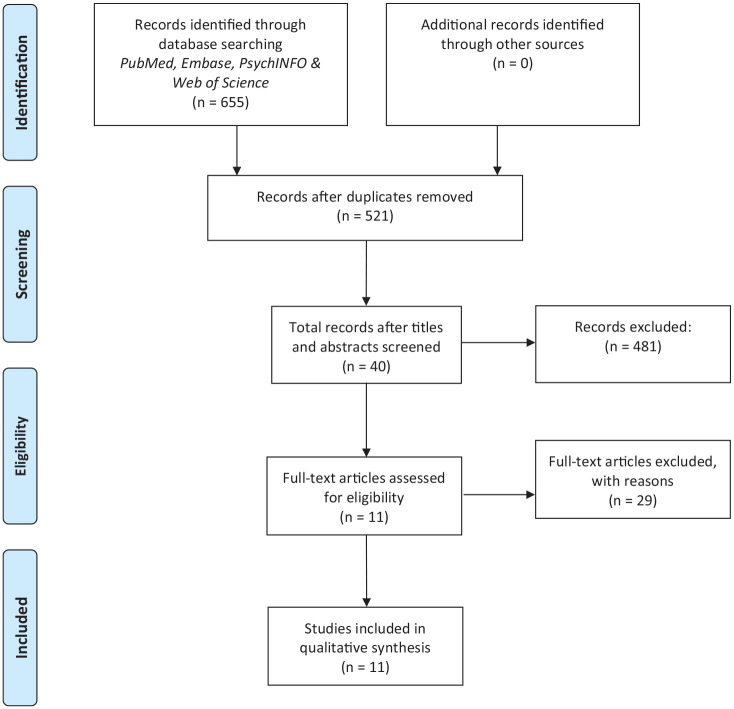
Inclusion of studies.

The electronic search (11th January 2021) yielded a total of 655 records (Web of Science: 135; PubMed: 158; Embase: 347; PsycINFO: 15). A total of 521 records remained after removal of duplicates. Then, the records were screened by title and abstract. Forty full-text records remained and were examined in detail. A total of 11 studies met the inclusion criteria and were included (Figure 1).

### Study characteristics

The 11 studies are summarized in Table 1.

**Table 1.** Summary of reviewed interventions.

Author, Study type	Study design	Sample	Active intervention	Control intervention	Primary outcome	Findings	Maintenance of effects	Study quality
Pharmacological								
Grima et al.,^30^ Group study	Randomized, placebo- controlled, double-blind, two-period, two-treatment crossover study	33 mild to severe TBI patients	**Melatonin group:** received daily 2 mg melatonin orally two hours before bedtime for four weeks	**Placebo group:** received daily 2 mg placebo capsule orally two hours before bedtime for four weeks	PSQI	Significant improvement in sleep quality	No follow-up assessment	Low risk of bias
					Actigraphy	No significant improvement in sleep onset latency		
Kemp et al.,^24^ Group study	Pilot, randomized	Seven mild to severe TBI patients	**Melatonin group:** patients received 5 mg melatonin for four weeks. Afterwards, patients followed the opposite therapy	**Amitriptyline group:** patients received 25 mg amitryptiline for four weeks. Afterwards, patients followed the opposite therapy	Sleep diary	No significant differences in sleep quality, duration, latency, or daytime alertness for either drug compared to baseline	No follow-up assessment	Some concerns
	Double-blind controlled crossover trial							
Behavioral								
Nguyen et al.,^25^ Group study	Pilot, randomized controlled trial	15 post-stroke patients	**CBT group:** patients received eight CBT sessions once weekly	**Treatment as usual group:** received usual care for eight weeks	FSS	Significant reduction in fatigue as compared to TAU	Effects at four months (follow-up) were maintained	Some concerns
Theadom et al.,^23^ Group study	Pilot, randomized controlled trial	24 mild or moderate TBI patients	**CBT group:** patients received six on-line CBT sessions once weekly	**Education control program:** patients received six on-line sessions once weekly. Patients received information about brain injury and sleep hygiene	PSQI	Significant improvement in self-reported sleep quality as compared to controls. No significant group differences on actigraphy measures	No follow-up assessment	Some concerns
					Actigraphy			
Nguyen et al.,^22^ Group study	Pilot, randomized controlled trial	24 mild to severe TBI patients	**CBT group:** patients received six modules across eight CBT sessions	**Treatment as usual group:** received usual care	PSQI	Significant improvements in sleep quality as compared to TAU	Effects maintained at two month follow-up	Some concerns
Ouellet and Morin,^19^ Single case	Single-case design	One moderate TBI patient with insomnia	The patient received eight CBT sessions once weekly		Sleep diary	Reductions in sleep onset latency, total time awake, and number of awakenings	Effects maintained at one and three month follow-up	Very high risk of bias
					Polysomnography	Improved sleep efficiency		
Ouellet and Morin,^20^ Single case	Single-case design	11 mild to severe TBI patients	Patients received eight CBT sessions once weekly		Sleep diary	Significant improvements in sleep efficiency	Effects maintained at one and three month follow-up	Substantial risk of bias
						Significant reductions in total wake time		
Herron et al.,^26^ Single case	Single-case design	Five stroke patients	Patients received seven individual modules over 7–12 weeks.		Sleep diary	Significant improved sleep duration in all patients	Effects maintained at two-week follow-up in three patients, except sleep duration	Very high risk of bias
						Significant improved sleep efficiency in three patients		
Other interventions								
Zollman et al.,^18^ Group study	Pilot study	24 chronic TBI patients	**Acupuncture group:** visits were twice a week for five weeks. Each acupuncture treatment lasted 20 minutes	**Control group:** control visits were twice a week for five weeks. Patients used their prescribed sleep medication	ISI	No significant differences in sleep time between the two groups	No follow-up assessment	Some concerns
					Actigraphy			
					HDRS			
					RBANS			
					PASAT	Acupuncture group showed a significant improvement in perception of sleep quality		
Huang et al.,^21^ Group study	Sham controlled randomized clinical trial	60 chronic mild TBI veterans	**Real acupuncture group:** received up to 10 sessions acupuncture over five weeks	**Sham acupuncture group:** received up to 10 sessions sham acupuncture over five weeks	PSQI	Significant improved sleep as compared to sham acupuncture group	No significant differences in sleep quality between the two groups at four-week follow-up	Low risk of bias
Chiu et al.,^17^ Group study	Feasibility randomized, controlled crossover trial	23 chronic TBI patients	**Home-based warm footbath group:** received a warm footbath (41) for three days. Afterwards, patients followed the opposite therapy	**Usual care group:** received usual care for three days. Afterwards, patients followed the opposite therapy	Actigraphy	Significant reduction in sleep onset latency and a shorter wake after sleep onset as compared to usual care	No follow-up assessment	Low risk of bias
						No significant improvements in sleep efficiency and total sleep time		

TBI: traumatic brain injury; PSQI: Pittsburgh Sleep Quality Index; CBT: cognitive behavioral therapy; ISI: Insomnia Severity Index; HDRS: Hamilton Depression Rating Scale; RBANS: repeatable battery for the assessment of neuropsychological status; PASAT: paced auditory serial addition test; FSS: Fatigue Severity Scale.

### Participant characteristics

A total of nine studies examined individuals with traumatic brain injury,^17–24^ and two studies examined stroke patients.^25,26^ The included studies involved 227 participants (207 individuals with traumatic brain injury, 20 stroke patients). One of the included studies recruited only participants with mild traumatic brain injury.^21^ All studies included adults with mean ages ranging from 27 to 60 years. The severity of traumatic brain injury was not reported in four of the group studies.^17,18,25,26^

### Outcome measures

Out of the 11 studies, 10 used subjective self-report sleep measurements as a primary outcome, including questionnaires on sleep quality such as the Pittsburgh Sleep Quality Index and questionnaires on the insomnia severity as measures by the Insomnia Severity Index. The most common was the self-reported sleep diary with sleep quality as the primary outcome.^19,20,24,26^ Three types of objective sleep assessments were used to gauge the effectiveness of the treatments for sleep disturbances. Actigraphy, a reliable and objective sleep measure, was the most common assessment of sleep quality.^17,18,23^ Actigraphy is a continuous measurement of sleep rhythms and wake periods via a recording device on the wrist.

### Risk of bias within studies

The Cochrane assessment of bias tool (RoB 2.0) was used to assess the quality of the included randomized controlled trials.^27^ The overall risk of bias is included in Table 1. With respect to the interventions aimed at pharmacological treatment, the study quality was mixed. One study was rated with “some concerns”^24^ and one study obtained a low risk of bias.^28^ The risk of bias in the studies aimed at behavioral treatment was rated with “some concerns” for all three studies.^22,23,25^ There was only a risk of bias related to the measurement of outcome because patients were aware of the treatment received. It is possible that this knowledge could have influenced their self-report on sleep quality following the intervention. However, the treatment as usual groups were also receiving active treatments in which patients also worked on their personal goals which diminishes this risk of bias. This diminished the risk of bias. Evaluating the study quality of the alternative interventions, two studies obtained a low risk of bias^17,21^ and one study was rated with “some concerns”.^18^

To rate the internal and external validity of the included single case studies, the Risk of Bias in N-of-1 trials (RoBiNT) was used.^29^ The overall risk of bias is included in Table 1. Two studies obtained a very high risk of bias^19,26^ and one study was classified as “substantial risk of bias”.^20^ A major limitation included inadequate sampling of the target behavior.

Table 1 provides a summary of the reviewed interventions, including descriptions of the methods, study designs, interventions, primary outcome measures, findings, maintenance of effects, and study quality.

### Pharmacological treatment

Two studies examined pharmacological treatment to improve sleep quality in patients with acquired brain injury.^24,30^ Pharmacological treatment included melatonin and amitriptyline. Melatonin intake (2 mg daily), which could be also considered as supplement, approximately two hours prior to bedtime, had a positive effect on sleep quality, but not on sleep onset latency in patients with traumatic brain injury.^30^ No follow-up assessment was conducted. This study obtained a low risk of bias with the use of a randomized controlled crossover design as a major strength. Kemp et al.^24^ compared the effect of melatonin (5 mg) with amitriptyline (25 mg) on sleep disturbances in individuals with mild to severe traumatic brain injury. Either melatonin or amitriptyline intake did not show improvements on sleep quality, sleep duration, sleep latency, or daytime alertness compared to baseline. No follow-up measurement was conducted. This study was rated with “some concerns”. There was a risk of bias in selection of the reported result as there was no information on a statistical analysis protocol or trial register.

### Cognitive Behavioral Therapy

All six cognitive behavioral therapy studies^19,20,22,23,25,26^ reported significant improvements in self-reported sleep quality. Two pilot randomized controlled trials examined the effect of cognitive behavioral therapy compared to treatment as usual on sleep quality in 15 stroke patients^25^ and in 24 individuals with mild to severe traumatic brain injury.^22^ In the first study, the cognitive behavioral therapy intervention consisted of eight sessions and addressed both fatigue and sleep in one intervention. Sleep interventions included stimulus control, bedtime restriction, hypersomnia management, relaxation, and cognitive restructuring. Sleep quality significantly improved in the cognitive behavioral therapy group compared to the treatment as usual group and the results maintained at four-month follow-up. Within the cognitive behavioral therapy group, four out of eight patients showed a clinically and significant improvement on the Pittsburgh Sleep Quality Index, which measures sleep quality. The second study found significant improvements in sleep quality in the cognitive behavioral group as compared to treatment as usual.^22^ Cognitive behavioral therapy included six modules: psychoeducation, sleep hygiene, cognitive restructuring, sleep interventions (stimulus control, bed restriction, and relaxation techniques), strategies for mental and physical fatigue, and relapse prevention. Nguyen et al. conducted a follow-up measurement and results showed that effects were maintained at two-month follow-up. Both pilot studies consist of a strong randomized controlled trial design, although conclusions are limited due to the small study sample. The study quality of both pilot studies was rated with “some” concerns. The only concern was that patients were aware of the treatment received and that this knowledge could have influenced their self-report on sleep quality. Therefore, there was a risk of bias in the measurement of outcome. Nevertheless, it is not very likely that the outcome was influenced by knowledge of the intervention as participants will not know the details and possible effects of the cognitive behavioral intervention compared to treatment as usual.

Another pilot randomized controlled trial examined the effect of cognitive behavioral therapy on sleep quality in 24 individuals with mild or moderate traumatic brain injury.^23^ Cognitive behavioral therapy consisted of psychoeducation, relaxation training, sleep restriction, cognitive therapy, information about the environment and sleep, and mindfulness meditation. The cognitive behavioral therapy group showed significant improvements in self-reported sleep quality as compared to an online education control group. Theadom et al.^23^ also used actigraphy, but they found no significant group differences on objective measures of sleep. No follow-up measurement was conducted. The cognitive behavioral intervention was administered online, which offers a cheaper and more accessible option. This study was rated with “some concerns”.^23^ There was no appropriate analysis used to estimate the effect of assignment to intervention. However, there was no potential for a substantial impact on the result of the failure to analyze participants in the group to which they were randomized.

All three pilot randomized controlled studies showed that cognitive behavioral therapy is an effective intervention for improvement of sleep quality in patients with acquired brain injury.^22,23,25^

In addition, three single case studies with a total of 17 patients with acquired brain injury (12 following traumatic brain injury and 5 post-stroke) reported positive effects of cognitive behavioral therapy for insomnia on several measures of sleep quality and sleep duration, using sleep diary reports^19,20,26^ and polysomnography.^19^ Cognitive behavioral therapy for insomnia interventions consisted of seven or eight sessions and included standard cognitive behavioral therapy for insomnia techniques such as psychoeducation, sleep hygiene, stimulus control, sleep restriction, relaxation, and cognitive restructuring. Clinically and significant improvements were found in 14 out of 17 patients. Effects were maintained at a follow-up assessment between two weeks and three months. The studies of Ouellet and Morin^20^ and Herron et al. obtained a very high risk of bias and one single case study was rated with a “substantial risk of bias”. One of the concerns was that treatment adherence was not assessed in the single case studies.

The cognitive behavioral interventions for treatment of sleep disturbances consisted of a combination of cognitive, behavioral, and educational components. Common and effective techniques were psychoeducation, cognitive restructuring, sleep hygiene, sleep interventions (bed restriction, stimulus control instructions and relaxation techniques), strategies for mental and physical fatigue, and relapse prevention.

### Other interventions

There are also studies that investigated treatments for insomnia using alternative approaches (i.e. non-pharmacological and no cognitive behavioral therapy for insomnia). Two controlled group studies examined the impact of acupuncture on sleep in 24 patients with chronic traumatic brain injury^18^ and in 60 veterans with mild traumatic brain injury.^21^ In the first study, participants in de acupuncture group received acupuncture at different points twice weekly for five weeks. Acupuncture was inserted on points in Kidney 3, Heart 3, Bladder 60, Liver 3, Large intestine, Pericardium 7, Governor Vessel 20, ear points Shen Men, and Tranquilizer. Each session lasted 20 minutes. The treatment regime was known as “standard dose” acupuncture protocol. Participants in the control group used their prescribed sleep medication. The acupuncture group showed significant improvement in the perception of sleep quality, but not on actigraphy total sleep time, as compared to controls.^18^ This study used a strong randomized controlled design and the risk of bias was low. However, a limitation is the lack of an active control group and no follow-up assessment. The second study compared a 10-session acupuncture treatment with a sham acupuncture treatment.^21^ Acupoints included standardized and individualized points. Dosage and treatment regime is not reported. The sham acupuncture group received sham needles without skin penetration. The acupuncture group showed larger improvements in sleep quality and on actigraphy measures posttreatment, however, these results were not maintained at follow-up. The study quality was rated with a low risk of bias.

Another alternative intervention comprised a home-based warm footbath aimed at improving sleep quality in 23 patients with traumatic brain injury and insomnia.^17^ This study is a randomized controlled trial with crossover design in which a three-day home-based warm footbath (41°C) intervention was compared to three days of usual care. The risk of bias was low. Sleep onset latency and shorter wake time after sleep onset (WASO), as measured with actigraphy, improved following the warm footbath compared to usual care. Sleep efficiency and total sleep time did not improve. Importantly, the positive effects of the warm footbath intervention were very small: sleep onset latency was shortened by 5.11 minutes. Furthermore, the effects diminish within days after patients stop taking the warm footbath in the evening.

## Discussion

### Summary of the evidence

The present systematic review aimed to provide an overview of the research on the treatments of sleep disturbances in individuals with acquired brain injury. Although it is clear that sleep disturbances following acquired brain injury are common and have a negative impact on daily functioning, the number of well-conducted studies regarding the treatment of sleep disturbances in acquired brain injury are limited.

To discuss the implications of the findings for clinical practice, we evaluated the results based on the study quality of the included studies. Studies were rated with a low risk of bias, with “some concerns,” or with a high risk of bias. For the treatment of sleep disturbances following acquired brain injury, two studies included pharmacological therapy,^24,30^ six studies examined the effects of cognitive behavioral therapy^19,20,22,23,25,26^ and three studies investigated alternative interventions^17,18,21^ such as acupuncture. Evidence for pharmacological therapy in patients with sleep disturbances due to acquired brain injury is scarce. Some positive effects of short-term melatonin were found in one study,^30^ however long-term effects are unknown. Furthermore, one study compared the effect of amitriptyline with melatonin but found no improvement in sleep quality for either drug.^24^

Effects of cognitive behavioral therapy were positive in all six studies up to a three- to four-month follow-up assessment.^19,20,22,23,25,26^ Reliable change analyses showed a clinically reliable improvement on sleep quality measures in 18 out of 25 acquired brain injury patients (72%).^19,20,25,26^ It should be noted that the current cognitive behavioral studies were either pilot studies^22,23,25^ or single case experimental design studies^19,20,26^ Although cognitive behavioral therapy has a potential in treating sleep disturbances following acquired brain injury with effects lasting up to several months, future studies are needed to examine the efficacy of cognitive behavioral therapy in well performed randomized controlled trial designs with larger samples and long-term follow up assessments.

Other investigated interventions included acupuncture and a home-based warm footbath. Two studies showed positive effects of acupuncture on self-reported sleep quality,^18,21^ however, these effects did not maintain at follow-up.^21^ Furthermore, Huang et al. recruited only participants with mild traumatic brain injury. The results of this study should therefore be interpreted with caution, as the findings cannot be generalized to the traumatic brain injury population at large. A home-based warm footbath (41°C) shortened the sleep onset latency and resulted in a shorter wake time after sleep onset, but the effects were very small and therefore not clinically relevant.^17^ Yet, it is a simple home-based intervention that, perhaps in combination with other interventions, could facilitate patients with acquired brain injury and sleep problems to fall asleep in the evening.

This review suggests that compared to pharmacological treatment, non-behavioral interventions seem an effective treatment for sleep disturbances following acquired brain injury. However, pharmacological agents are routinely prescribed by clinicians and especially administration of short-term medication is still the treatment of choice for sleep disturbances following traumatic brain injury and stroke.^31,32^ However, pharmacological agents can induce sedation, tolerance, and dependence.^33^ Since pharmacological interventions are still recommended for treatment of sleep disturbances following acquired brain injury, regardless of their side-effects, it is of paramount importance to evaluate and compare the effectiveness of both pharmacological and non-pharmacological interventions directly for treatment of sleep disturbances in acquired brain injury patients.

In the general population, cognitive behavioral therapy is recommended for the treatment of sleep disturbances.^34,35^ Compared to pharmacological treatments, cognitive behavioral therapy has the same beneficial effects on short-term and better long-term effects than benzodiazepines or benzodiazepine receptor agonists.^36,37^ Melatonin and acupuncture are not recommended due to low quality evidence and only short-term effects.^34,35^ Although the evidence concerning treatment of insomnia in the acquired brain injury population is not as well established, the results of this systematic review are in line with the clinical guidelines for sleep disturbances in the general population.

Although cognitive behavioral therapy is recommended in the general population for treatment of sleep disturbances, there appears to be circumstances where cognitive behavioral therapy is not feasible (e.g. the cognitive character of the therapy may be inappropriate for individuals with severe traumatic brain injury and severe cognitive impairment). Then, pharmacological therapies, acupuncture, or perhaps a simple home-based warm footbath may be more appropriate alternatives. However, future trials should further investigate the efficacy of these interventions and especially in individuals with severe brain injuries to draw stronger conclusions.

### Study strengths and limitations

To our knowledge, this is the first systematic review providing a general overview including both pharmacological and non-pharmacological treatments for sleep disturbances following acquired brain injury. A strength is that we appraised the quality of the studies using the revised Cochrane assessment of bias tool, a valid tool for rating the risk of bias. Taking study quality into account, strengthens the value of clinical recommendations made. Moreover, this systematic review focused on treatments for sleep disturbances following acquired brain injury. Most studies require merely self-reported sleep disturbances as an inclusion criterium and did not diagnose a sleep disorder according to international standards.^17,18,21–25,28^ By focusing on sleep disturbances including problems with sleep onset and maintenance, we reached a broader group of patients. Individuals with sleep disturbances experience difficulties in everyday functioning and should be able to receive treatment, without meeting all diagnostic criteria for a formal sleep disorder.^1^

However, the present review also has a number of limitations. First, the objective of this systematic review was to provide an overview including both pharmacological and non-pharmacological interventions for treatment of sleep disturbances following acquired brain injury (three months post-injury). As a consequence, this review excluded studies with individuals in the (sub)acute phase of acquired brain injury (<three months post-injury). We are therefore unable to determine the extent to which the findings generalize to samples of individuals with sleep problems and acquired brain injury in the acute phase of injury since the type of sleep disturbances can vary considerably per phase of injury.^38,39^ Second, in order to maintain a certain quality concerning the study designs, this review only included controlled group and single case experimental studies, resulting in a relatively small number of included studies. Despite the selection on designs, the quality of the studies was mixed. As a result of the limited quantity and quality of the studies, a meta-analysis could not be performed. Because of the limited number of studies and the variation in quality, the conclusions of this review may be considered global. Third, in the context of this review, acquired brain injury included traumatic brain injury and stroke. Only two studies (both including cognitive behavioral therapy) focused on stroke patients.^25,26^ Nevertheless, cognitive behavioral therapy has a promising potential in treating sleep disturbances following acquired brain injury. However, other interventions included only participants with traumatic brain injury which means that findings of these interventions are limited to the traumatic brain injury population. Finally, it should be noted that only two studies examined pharmacological treatment to improve sleep quality. In fact, none of the pharmacological studies investigated the effect of benzodiazepines on sleep quality. Clinical guidelines, however, still recommend benzodiazepines for treatment of sleep disturbances.^34^ One study examined the effect of benzodiazepines in persons with acquired brain injury from an inpatient rehabilitation ward.^28^ However, the time since injury of the included participants is unknown and therefore outside the scope of this review. Shan and Ashworth^28^ compared the effect of lorazepam (0.5–1.0 mg daily) with zopiclone (3.75–7.5 mg daily) on sleep duration in patients with acquired brain injury and insomnia. Results showed no difference between the two interventions. This study was rated with a high risk of bias as it did not have a placebo arm, did not use validated measures of sleep quality and did not include baseline measures. A major limitation included a high risk of bias in the outcome measurement, which consisted of recording the total sleep time as measured by the nursing staff. Based on the study design, no conclusions could be drawn regarding the effect of lorazepam or zopiclone on sleep quality in patients with acquired brain injury. The limited number of studies examining pharmacological treatment of sleep disturbances following acquired brain injury, preclude us from drawing confirmative conclusions.

## Conclusions

Although there was heterogeneity in the study quality of the included studies, their outcomes suggest that cognitive behavioral therapy is recommended as treatment of choice for sleep disturbances following acquired brain injury, with positive short-and long-term effects on sleep duration and sleep quality. Since other interventions included only participants with traumatic brain injury, findings of these interventions are therefore limited to the traumatic brain injury population. A cognitive behavioral intervention of eight sessions including standard techniques such as psychoeducation, sleep hygiene, cognitive restructuring, sleep interventions (stimulus control, bed restriction, and relaxation techniques) and relapse prevention, is recommended for individuals with mild to severe traumatic brain injury and stroke in the chronic phase of recovery (range 25–48 months post-injury). Furthermore, an online form of cognitive behavioral therapy seems to be an effective method for patients with traumatic brain injury, which is increasingly available for patients and a cost-effective intervention.

With respect to future research in this area, more research of higher quality should further examine long-term effects of (cognitive) behavioral sleep therapies as well as their specific predictive variables of treatment success.

**Clinical messages**

- Cognitive behavioral therapy is recommended for treatment of sleep disturbances in patients with acquired brain injury
- Six to eight sessions of cognitive behavioral therapy in patients with mild to severe traumatic brain injury improves sleep quality

## Supplemental Material

sj-pdf-1-cre-10.1177_02692155211014827 – Supplemental material for Treatments for sleep disturbances in individuals with acquired brain injury: A systematic review Click here for additional data file. Supplemental material, sj-pdf-1-cre-10.1177_02692155211014827 for Treatments for sleep disturbances in individuals with acquired brain injury: A systematic review by Louise Pilon, Nikita Frankenmolen and Dirk Bertens in Clinical Rehabilitation
