# Supplementary material for: Treatments for sleep disturbances in individuals with acquired brain injury: A systematic review
Source: Clin Rehabil. 2021 May 20;35(11):1518–29. doi: 10.1177/02692155211014827 (PMC8524684; doi:10.1177/02692155211014827)
Supplement: sj-pdf-1-cre-10.1177_02692155211014827 – Supplemental material for Treatments for sleep disturbances in individuals with acquired brain injury: A systematic review [file sj-pdf-1-cre-10.1177_02692155211014827.pdf]

Supplemental Material

Table S1. Search strategy for PubMed Complete.

S1	“Sleep initiation and Maintenance Disorders” [Mesh] OR “Sleep Wake Disorders” [Mesh] OR Insomnia*[tiab] OR Sleeplessness*[tiab] OR sleep difficult*[tiab] OR sleep deprivati*[tiab] OR sleep disturbance*[tiab] OR sleep wake disorder*[tiab] OR dyssomnia*[tiab]	113101
S2	“Brain Injuries"[Mesh] "Stroke"[Mesh]OR "Brain Ischemia"[Mesh] OR "Head Injuries, Closed"[Mesh] OR "Head Injuries, Penetrating"[Mesh] OR brain injur*[tiab] OR stroke*[tiab] OR brain damag*[tiab] OR head injur*[tiab] OR cerebrovascular accident*[tiab] OR brain infarct*[tiab] OR cerebral infarct*[tiab] OR brain lesion*[tiab] OR ischemic lesion*[tiab] OR cerebral ischemia*[tiab]	447495
S3	S1 AND S2	3057
S4	"Sleep Initiation and Maintenance Disorders"[Mesh] OR "Sleep Wake Disorders"[Mesh] OR Insomnia*[tiab] or Sleeplessness*[tiab] or sleep difficult*[tiab] or sleep deprivati*[tiab] or sleep disturbanc*[tiab] or sleep wake disorder*[tiab] or dyssomnia*[tiab]) AND ("Brain Injuries"[Mesh] "Stroke"[Mesh]OR "Brain Ischemia"[Mesh] OR "Head Injuries, Closed"[Mesh] OR "Head Injuries, Penetrating"[Mesh] OR brain injur*[tiab] OR stroke*[tiab] OR brain damag*[tiab] OR head injur*[tiab] OR cerebrovascular accident*[tiab] OR brain infarct*[tiab] OR cerebral infarct*[tiab] OR brain lesion*[tiab] OR ischemic lesion*[tiab] OR cerebral ischemia*[tiab] AND (Therapy/Narrow[filter] OR single-case[Title/Abstract])	158

Table S2. Search strategy for Embase Complete.

S1	exp insomnia/	70301
S2	exp sleep disorder/	246751
S3	(Insomnia* or Sleeplessness* or sleep difficult* or sleep deprivati* or sleep disturbanc* or sleep wake disorder* or dyssomnia*).ti,ab,kw.	75868
S4	exp brain injury/	184100
S5	1 or 2 or 3	266472
S6	exp cerebrovascular accident/	221361
S7	exp brain ischemia/	193179
S8	exp head injury/	297058
S9	(brain injur* or stroke* or brain damag* or head injur* or cerebrovascular accident* or brain infarct* or cerebral infarct* or brain lesion* or ischemic lesion* or cerebral ischemia*).ti,ab,kw.	613262
S10	4 or 6 or 7 or 8 or 9	874210
S11	5 and 10	13132
S12	((randomized and controlled and trial) or single-case).ti,ab.	213781
S13	11 and 12	347

Table S3. Search strategy for PsycInfo Complete.

1	"sleep initiation and maintenance disorders".mh.	4563
2	"sleep wake disorders".mh.	6422
3	(Insomnia* or Sleeplessness* or sleep difficult* or sleep deprivati* or sleep disturbanc* or sleep wake disorder* or dyssomnia*).ti,ab,id.	25505
4	("brain injuries" or stroke or "brain ischemia" or "head injuries, closed" or "head injuries, penetrating").mh.	25037
5	1 or 2 or 3	29853
6	("brain injur*" or stroke* or "brain damag*" or "head injur*" or "cerebrovascular accident*" or "brain infarct*" or "cerebral infarct*" or "brain lesion*" or "ischemic lesion*" or "cerebral ischemia*").ti,ab,id.	84132
7	4 or 6	86982
8	5 and 7	754
9	((randomized and controlled and trial) or single-case).ti,ab.	34517
10	8 and 9	15

Table S4. Search strategy for Web of Science Complete.

S1	TS=(Insomnia* OR Sleeplessness* OR "sleep difficult*" OR "sleep deprivati*" OR "sleep disturbanc*" OR "sleep wake disorder*" OR dyssomnia*)<br>*Indexes=SCI-EXPANDED, SSCI, A&HCI, CPCI-S, CPCI-SSH, BKCI-S, BKCI-SSH, ESCI, CCR-EXPANDED, IC Timespan=All years*	58357
S2	TS=("brain injur*" OR stroke* OR "brain damag*" OR "head injur*" OR "cerebrovascular accident*" OR "brain infarct*" OR "cerebral infarct*" OR "brain lesion*" OR "ischemic lesion*" OR "cerebral ischemia*")<br>*Indexes=SCI-EXPANDED, SSCI, A&HCI, CPCI-S, CPCI-SSH, BKCI-S, BKCI-SSH, ESCI, CCR-EXPANDED, IC Timespan=All years*	547515
S3	TS=(randomized AND controlled AND trial OR single-case)<br>*Indexes=SCI-EXPANDED, SSCI, A&HCI, CPCI-S, CPCI-SSH, BKCI-S, BKCI-SSH, ESCI, CCR-EXPANDED, IC Timespan=All years*	450823
S4	#3 AND #2 AND #1<br>*Indexes=SCI-EXPANDED, SSCI, A&HCI, CPCI-S, CPCI-SSH, BKCI-S, BKCI-SSH, ESCI, CCR-EXPANDED, IC Timespan=All years*	135
